# The cGAS-STING pathway-dependent sensing of mitochondrial DNA mediates ocular surface inflammation

**DOI:** 10.1038/s41392-023-01624-z

**Published:** 2023-09-21

**Authors:** Weijie Ouyang, Shoubi Wang, Dan Yan, Jieli Wu, Yunuo Zhang, Wei Li, Jiaoyue Hu, Zuguo Liu

**Affiliations:** 1 Xiamen University affiliated Xiamen Eye Center; Fujian Provincial Key Laboratory of Ophthalmology and Visual Science; Fujian Engineering and Research Center of Eye Regenerative Medicine; Eye Institute of Xiamen University; School of Medicine, Xiamen University, Xiamen, Fujian 361005 China; 2 Department of Ophthalmology, Xiang’an Hospital of Xiamen University, Xiamen, Fujian 361005 China; 3 The First Affiliated Hospital of Xiamen University, School of Medicine, Xiamen University, Xiamen, 361005 China; 4 Changsha Aier Eye Hospital, Changsha, Hunan 410016 China; 5 Department of Ophthalmology, The First Affiliated Hospital of University of South China, Hengyang, Hunan 421001 China

**Keywords:** Immunological disorders, Innate immunity

## Abstract

The innate immune response is the main pathophysiological process of ocular surface diseases exposed to multiple environmental stresses. The epithelium is central to the innate immune response, but whether and how innate immunity is initiated by ocular epithelial cells in response to various environmental stresses in ocular surface diseases, such as dry eye, is still unclear. By utilizing two classic experimental dry eye models—a mouse ocular surface treated with benzalkonium chloride (BAC) and a mouse model with surgically removed extraorbital lachrymal glands, as well as dry eye patient samples—along with human corneal epithelial cells (HCE) exposed to hyperosmolarity, we have discovered a novel innate immune pathway in ocular surface epithelial cells. Under stress, mitochondrial DNA (mtDNA) was released into the cytoplasm through the mitochondrial permeability transition pore (mPTP) and further activated the cyclic GMP-AMP synthase (cGAS)—stimulator of interferon genes (STING) pathway, aggravating downstream inflammatory responses and ocular surface damage. Genetic deletion or pharmacological suppression of STING and inhibition of mtDNA release reduced inflammatory responses, whereas mtDNA transfection supported cytoplasmic mtDNA-induced inflammatory responses by activating the cGAS-STING pathway. Our study clarified the cGAS-STING pathway-dependent sensing of mitochondrial DNA-mediated ocular surface inflammation, which elucidated a new mechanism of ocular surface diseases in response to multiple environmental stresses.

## Introduction

Recently, the concept of innate immunity evolved to encompass the recognition of environmental pathogens beyond microbial components. Growing evidence shows that the activation of the innate immune response has been found to participate in many noninfectious inflammatory diseases^1–4^ triggered by various stresses or damage-induced danger-associated molecular patterns (DAMPs).^1,5–9^ With recent breakthroughs, the therapeutic targeting of innate immunity has emerged as a viable approach for treating inflammatory diseases, such as dry eye,^10^ acute kidney injury (AKI),^11^ hepatitis^12^ and autoinflammatory bone disorders.^13^ Furthermore, epithelial cells are reported to recognize and participate in innate immune responses, which hold a crucial function in immunomodulation.^8,14^

On the ocular surface, the epithelium is central to the innate immune response.^15^ Corneal epithelial cells reside in the outermost layer of the ocular surface and are in direct contact with the environment.^16^ Long-term exposure to multiple environmental stresses, including low humidity, high wind velocity, blue light and preservatives, could increase tear film instability and hyperosmolarity, leading to epithelial cell damage and inflammatory responses and eventually ocular surface diseases,^17,18^ among which dry eye is the most prominent.^19,20^ Ocular surface inflammation is a hallmark of epithelial cell stresses, which are generated by the innate immune system.^21^ Immunomodulatory therapies targeting chronic inflammation has been shown to be effective on ocular surface diseases. However, the extent to which ocular epithelial cells initiate innate immunity in response to diverse environmental stressors remains largely ambiguous, and clarifying this issue will be helpful to better guide the treatment of ocular surface diseases.

The cyclic GMP-AMP synthase (cGAS)- stimulator of interferon genes (STING) pathway is an inflammatory signaling pathway discovered in recent years by recognition of cytoplasmic DNA.^22–24^ Under various stress conditions, the DNA from the nucleus and mitochondria release into the cytoplasm.^25^ cGAS recognizes cytoplasmic double-stranded DNA (dsDNA) and produces a second messenger known as 2’3’-cGAMP, which further activates the expression and translocation of STING, inducing the phosphorylation of TBK1 (p-TBK1) and IRF3 (p- IRF3) to further mediate the release of downstream inflammatory cytokines (IFN-a/β and CXCL10).^26^ Studies has indicated that the activation of the cGAS-STING pathway is observed in age-dependent macular degeneration (AMD),^27^ Parkinson’s disease,^28^ alcoholic liver disease,^29^ nonalcoholic steatohepatitis,^30,31^ atherogenesis^32^ and AKI,^33^ and pharmacological inhibition of STING can effectively improve the progression of inflammatory diseases, which indicates that the cGAS-STING pathway may have therapeutic potential in autoimmune and inflammatory diseases. However, the role of the cGAS-STING pathway in ocular surface diseases exposed to various environmental stresses remains largely unexplored.

In a previous study, we speculated that mitochondrial DNA released into the cytoplasm may cause inflammation via the cGAS-STING signaling pathway under hyperosmotic stress (HS).^34^ In this study, we have provided evidence for the activation of the cGAS-STING pathway by mitochondrial DNA sensing, which mediates ocular surface inflammation in two experimental dry eye models—a mouse ocular surface treated with BAC and a mouse model with surgically removed lacrimal glands, as well as dry eye patient samples—along with cultured HCE exposed to hyperosmotic stress (HS-HCE). This study represents the first investigation of this novel pathway in ocular surface diseases, offering a new perspective on inflammatory responses occurring at the ocular surface. The identification of this pathway, associated with the innate immune response, may open up potential therapeutic targets for ocular surface diseases.

## Results

### Activation of the cGAS-STING pathway was observed in BAC-induced ocular surface damage in mice and dry eye patients

**Fig. 1:**
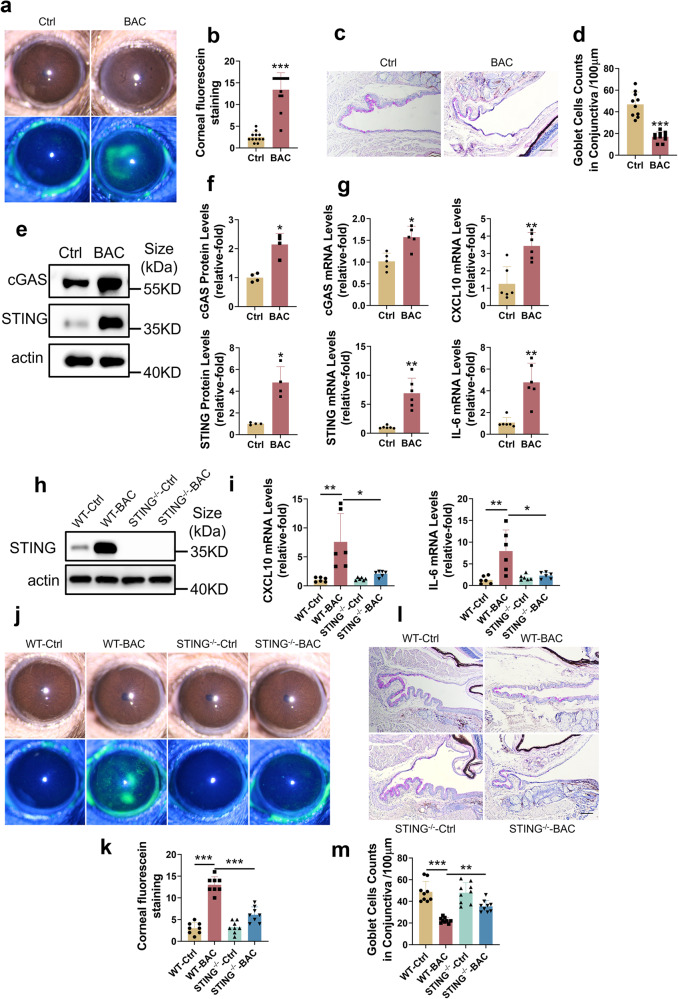
Activation of the cGAS-STING pathway was associated with BAC-induced ocular surface damage in mice. **a**, **b** Increased CFS scores in BAC-treated mice. Representative images of CFS (**a**) and mean scores (**b**) in normal control and BAC-treated mice were shown. **c**, **d** Decreased goblet cells in BAC-treated mice. Representative images of PAS staining (**c**) and mean numbers (**d**) in normal control and BAC-treated mice were shown. Scale bars: 100 μm. **e**, **f** cGAS and STING protein levels were increased in BAC-treated mice (*n* = 3). **g** mRNA expression of cGAS, STING and inflammatory cytokines, including CXCL10 and IL-6, was increased in BAC-treated mice (*n* = 6). **h** The expression of STING in both WT and STING KO mice with or without BAC induction. **i** Upregulation of IL-6 and CXCL10 mRNA in BAC-treated mice was attenuated in STING KO mice (*n* = 6). **j**, **k** BAC-induced CFS scores were reversed in STING KO mice. Representative images of CFS (**j**) and mean scores (**k**) were shown**. l**, **m** BAC-induced goblet cell loss was reversed in STING KO mice. Representative images of PAS staining (**l**) and mean numbers (**m**). Scale bars: 100 μm. The data were shown as the mean ± SD. *P < 0.05, **P < 0.01, ***P < 0.001

BAC-induced ocular surface damage is a frequently employed disease model of the ocular surface.^35^ In our study, BAC-treated mice developed widespread corneal fluorescein staining (CFS) accompanied by goblet cell loss on Day 7 (Fig. 1a–d). In addition, increased levels of cGAS and STING (Fig. 1e–g) and their downstream inflammatory cytokines (Fig. 1g) were observed in BAC-treated mice. We also found activation of the cGAS-STING pathway in mice with extraorbital lachrymal gland excision (Supplementary Fig. 1a–e), another tear deficiency-induced ocular surface damage model.^36^

Dry eye is the most common chronic inflammatory disease of the ocular surface,^19^ and the inflammatory response has been identified as the central basis of dry eye, irrespective of its underlying causes.^37,38^ In order to investigate the activation of the cGAS-STING pathway in dry eye, we extracted tear proteins from Schirmer strips of both dry eye patients and normal individuals (Supplementary Fig. 2a). Notably, the activation of the cGAS-STING pathway was activated in dry eye patients. As shown in Supplementary Fig. 2b, c, the protein levels of cGAS and STING were increased in dry eye patients. In addition, our findings showed a positive correlation between the levels of STING protein and the severity of CFS scores (rs = 0.692, *P* = 0.001) as well as a negative correlation with the Schirmer I test (rs = −0.340, *P* = 0.142) (Supplementary Fig. 2d). Overall, these results provide evidence of the activation of the cGAS-STING pathway in both mouse models of ocular surface disease and dry eye patients.

### Genetical or pharmacological suppression of STING ameliorated BAC-induced ocular surface damage in mice

To confirm the role of the cGAS-STING pathway in ocular surface damage, STING knockout (STING^-/-^) mice were utilized. STING was increased in BAC-treated WT mice but was absent in STING^-/-^ mice (Fig. 1h). BAC-induced widespread CFS and goblet cell loss were significantly attenuated in STING^-/-^ mice (Fig. 1j–m), with downregulation of inflammatory cytokines (Fig. 1i). Taken together, the cGAS-STING pathway was involved in BAC-induced ocular surface damage and associated inflammation.

**Fig. 2:**
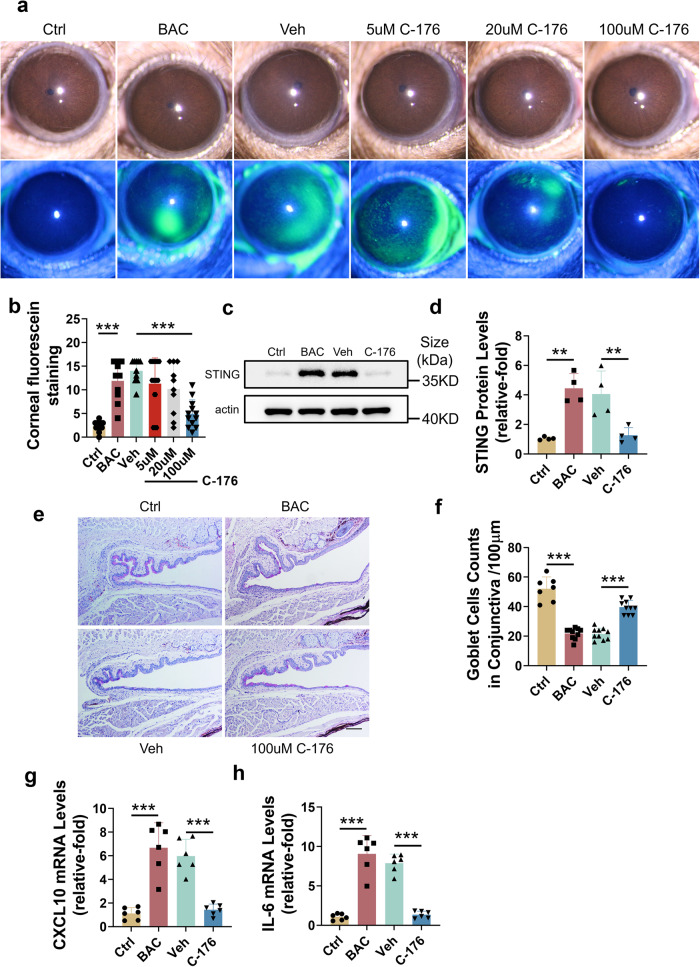
Pharmacological suppression of STING ameliorated BAC-induced ocular surface damage in mice. **a**, **b** C-176 ameliorated BAC-induced CFS. Representative images of CFS (**a**) and mean scores (**b**) were shown. **c**, **d** BAC-induced STING expression was reversed by C-176 treatment (*n* = 3). **e**, **f** BAC-induced goblet cell loss was reversed by C-176 treatment. Representative images of PAS staining (**e**) and mean numbers (**f**). Scale bars: 100 μm. **g**, **h** Upregulation of CXCL10 (**g**) and IL-6 (**h**) mRNA in BAC-induced dry eye mice was attenuated by C-176 treatment (*n* = 6). The data were shown as the mean ± SD. **P < 0.01, ***P < 0.001

To further explore the role of STING in BAC-induced ocular damage, C-176,^39^ a pharmacological inhibitor of STING, was applied to BAC-treated mice. Different concentrations of C-176 (1–100 µM) were applied four times a day. Only 100 µM C-176 significantly ameliorated BAC-induced corneal epithelial damage (Fig. 2a, b). In addition, 100 µM C-176 significantly inhibited the activation of STING (Fig. 2c, d) and decreased the BAC-induced loss of goblet cells (Fig. 2e, f) as well as the levels of inflammatory cytokines (Fig. 2g, h). These results showed that pharmacological inhibition of STING had great potential for the treatment of ocular surface damage.

### cGAS-STING pathway activation was associated with HS-induced inflammation

**Fig. 3:**
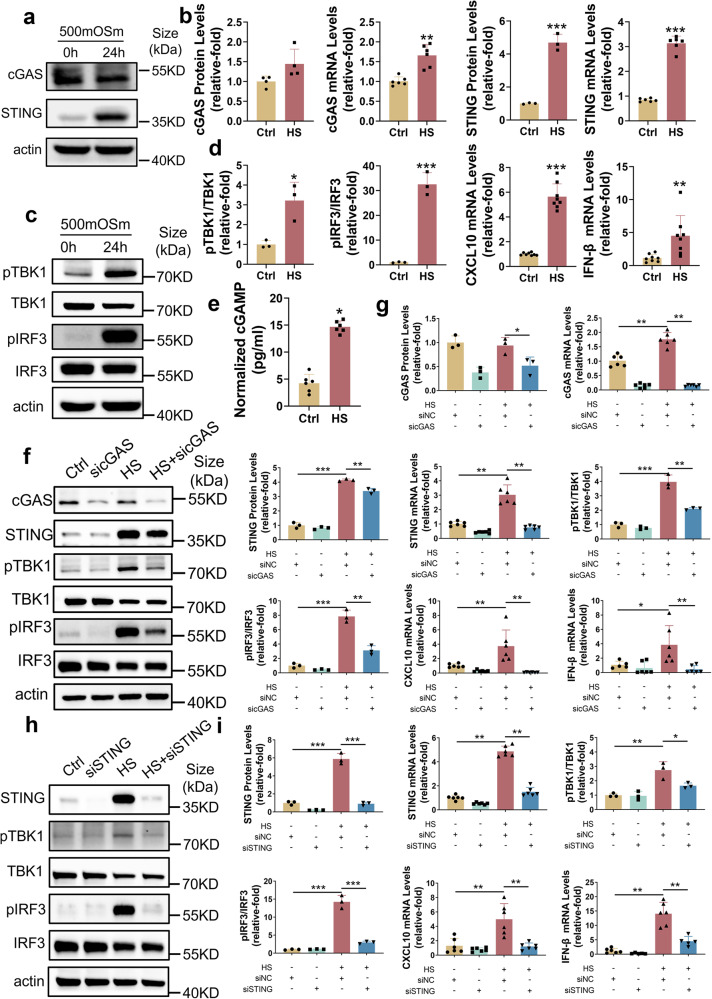
Inflammation was associated with activation of the cGAS-STING pathway under hyperosmotic stress**. a**, **b** Hyperosmotic stress increased the protein and mRNA levels of cGAS and STING. **c**, **d** The downstream molecules of STING were activated under HS. The p-TBK1 and p-IRF3 was upregulated under HS (**c**). The expression levels of the inflammatory cytokines CXCL10 and IFN-β were upregulated under HS (**d**). **e** cGAMP levels were increased under HS for 24 h (*n* = 6). **f**, **g** siRNA-mediated cGAS knockdown suppressed HS-induced p-TBK1 and p-IRF3 and HS-induced upregulation of CXCL10 and IFN-β (*n* = 3 or 6). **h**, **i** siRNA-mediated STING knockdown suppressed HS-induced p-TBK1 and p-IRF3 and HS-induced upregulation of CXCL10 and IFN-β (*n* = 3 or 6). The data were shown as the mean ± SD. *P < 0.05, **P < 0.01, ***P < 0.001

HS is one of the most common ocular surface stresses since environmental challenges can increase tear hyperosmolarity.^40^ Hence, we further evaluated the role of the cGAS-STING pathway in HS-HCE. The cGAS-STING pathway was activated when exposed to HS (500 OSmO, 24 h) (Fig. 3a, b). Although there was no difference in cGAS protein levels between the Ctrl and HS groups, the mRNA levels of cGAS (Fig. 3b) and cGAMP (Fig. 3e) were increased under HS conditions, which directly indicates activation of cGAS. Simultaneously, p-TBK1, p-RF3 (Fig. 3c, d) and inflammatory cytokines (CXCL10 and IFN-β) were upregulated (Fig. 3d) under HS exposure.

To demonstrate the role of the cGAS-STING pathway, small interfering RNA (siRNA) was transferred into HCE to knockdown cGAS or STING, which further led to a decline in HS-induced p-TBK1, p-IRF3 (Fig. 3f–i), CXCL10 and IFN-β (Fig. 3g, i). Taken together, these results demonstrated that the cGAS-STING pathway contributed to HS-induced inflammation.

### Cytoplasmic mtDNA activated the cGAS-STING pathway in HCE under HS exposure

**Fig. 4:**
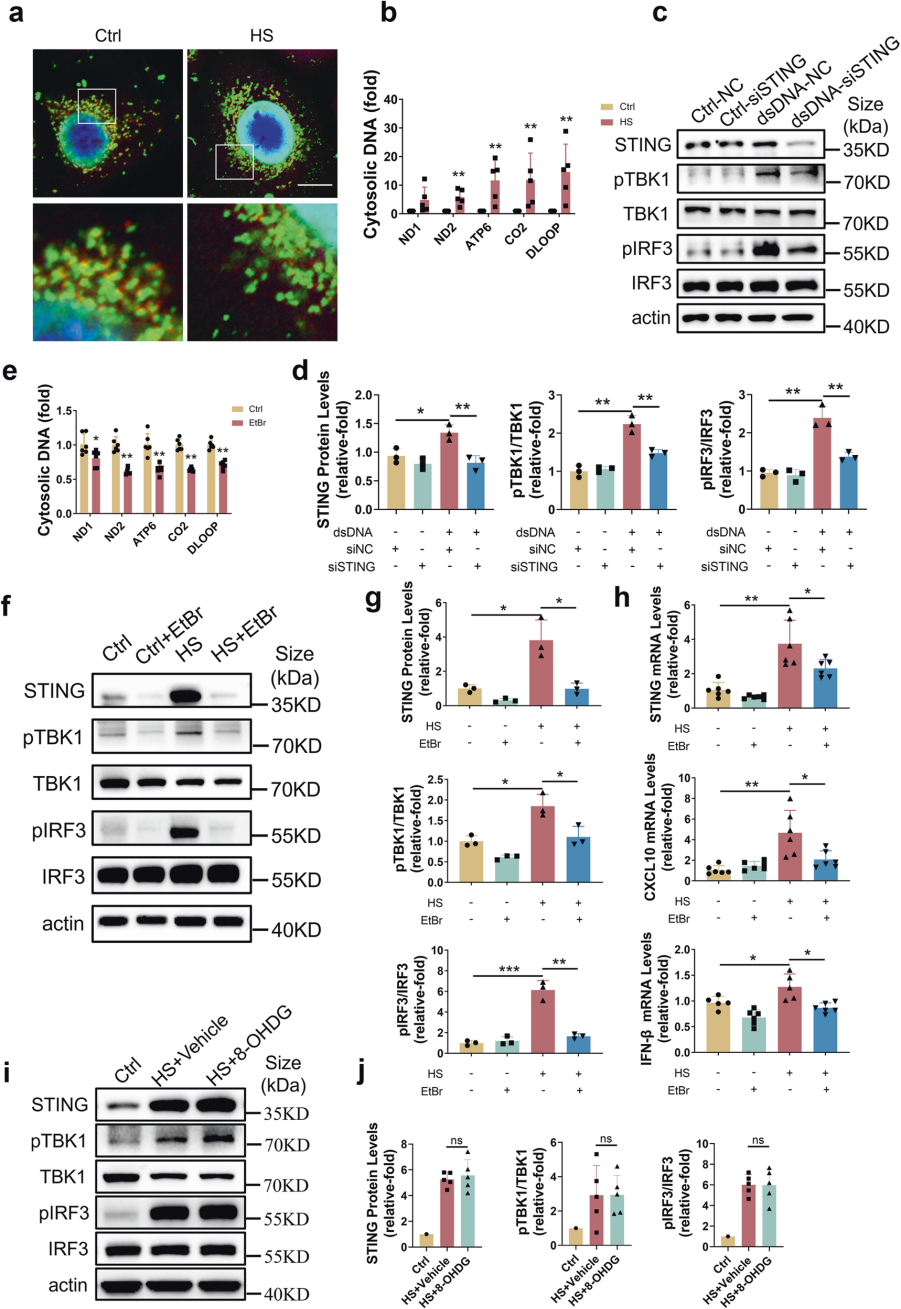
mtDNA leakage into the cytosol mediated the cGAS-STING pathway activation under hyperosmotic stress**. a** Representative fluorescent micrographs of Ctrl and HS groups stained with dsDNA (green) and mitochondria (red). Image on the lower portion represents an enlarged section of the square frame in the upper portion. Scale bars: 10 μm. **b** Cytoplasmic mtDNA copy number was determined by real-time PCR (*n* = 5). **c**, **d** siRNA-mediated STING knockdown blocked mtDNA-induced p-TBK1 and p-IRF3 (*n* = 3). **e** Cytoplasmic mtDNA copy number was determined by real-time PCR after treatment for 48 h with EtBr (0.2 mg/ml) (*n* = 6). **f**–**h** HS-induced activation of STING, p-TBK1 and p-IRF3 and upregulation of CXCL10 and IFN-β were reversed by EtBr-mediated mtDNA depletion (*n* = 3 or 6). **i**, **j** 8-OHDG did not ameliorate HS-induced increases in STING levels or p-TBK1 and p-IRF3. The data were shown as the mean ± SD. *P < 0.05, **P < 0.01, ***P < 0.001

dsDNA, including mtDNA released into the cytoplasm and oxidized mtDNA, is the initial factor for activating the cGAS-STING pathway.^23,24^ To elucidate the mechanism of cGAS-STING pathway activation on the ocular surface, we further detected the expression of mtDNA. dsDNA was observed to be translocated into the cytosol in HS-HCE (Fig. 4a). The quantified mtDNA results using mtDNA-specific PCR showed that cytosolic mtDNA was significantly increased under HS exposure (Fig. 4b). To demonstrate whether cGAS-STING pathway activation was associated with mtDNA leakage into the cytosol in HS-HCE, we isolated mtDNA and transfected it into HCE. Transfected cytosolic mtDNA (1 µg/ml) rescued the p-TBK1 and p-IRF3, which was inhibited by siSTING (Fig. 4c, d). In addition, mtDNA was depleted from HCE by ethidium bromide (EtBr), a compound that has been used to block the replication of mtDNA rather than nuclear DNA at low concentrations (0.1–2 μg/mL).^41^ The mtDNA copy number was inhibited by approximately 50% in HCE cultured with EtBr (0.2 µg/ml) for two days (Fig. 4e). Moreover, HS-induced activation of STING, p-TBK1 and p-IRF3 and upregulation of inflammatory cytokines (CXCL10 and IFN-β) were inhibited in HCE with EtBr treatment (Fig. 4f–h). Oxidized mtDNA, which has been observed in HS-HCE,^40^ also activates the cGAS-STING pathway.^42^ In our study, exogenous 8-OHdG, which could block oxidized mtDNA, did not inhibit the cGAS-STING pathway (Fig. 4i, j). These results indicated that HS induced the release of mtDNA rather than oxidized mtDNA into the cytosol to activate the cGAS-STING pathway.

### mPTP opening mediated mtDNA leakage and cGAS-STING pathway activation under HS exposure

**Fig. 5:**
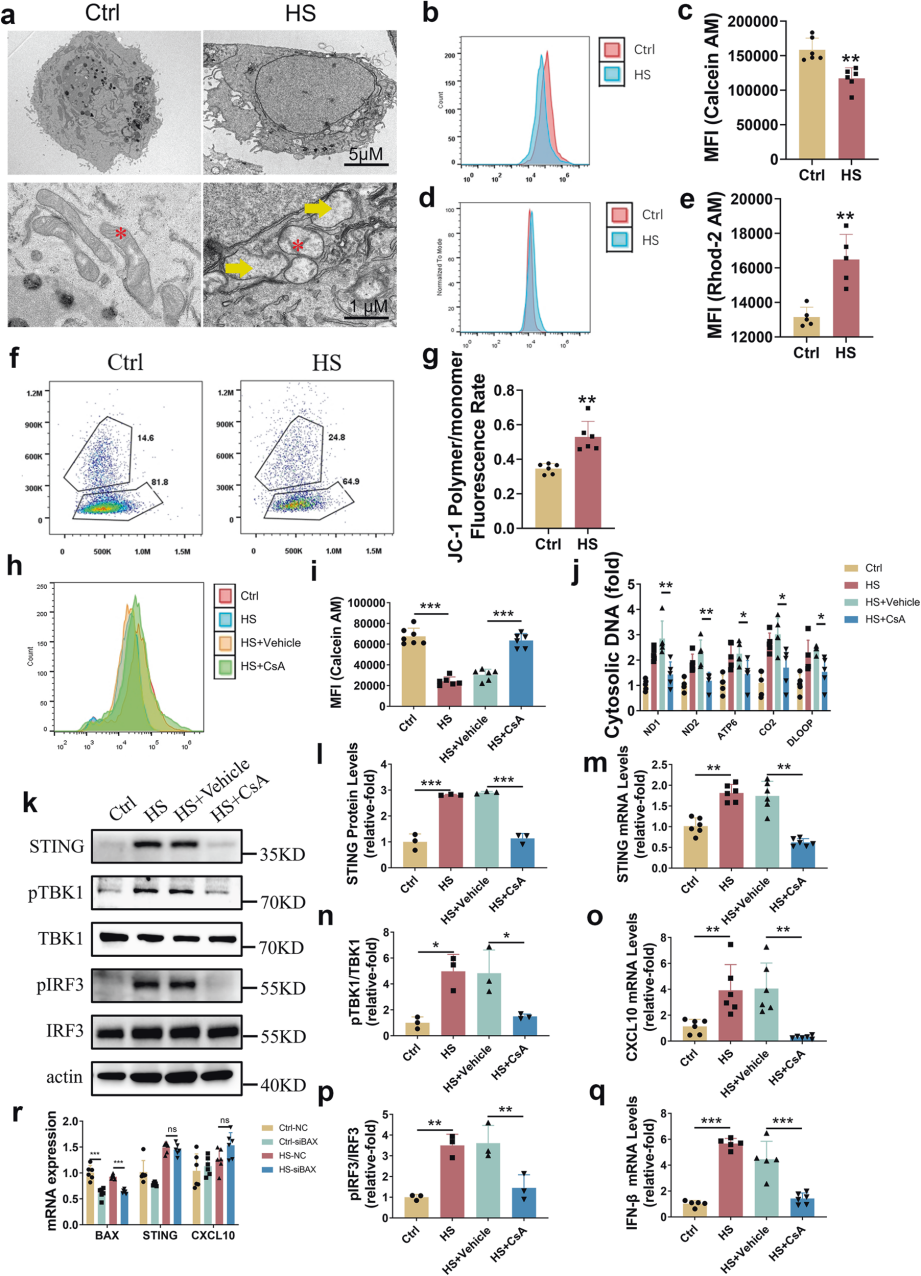
mPTP opening mediated mtDNA leakage and cGAS-STING pathway activation under hyperosmotic stress. **a** Transmission electron microscopy images of HCE with or without HS. HS induced loss of mitochondrial cristae (red asterisk) and swelling in the mitochondrial matrix (yellow arrow). **b**, **c** HS induced mPTP opening. The mPTP level was determined by flow cytometry (**b**), and the mean fluorescein index (MFI) was shown (**c**). **d**, **e** HS increased mitochondrial Ca^2+^ levels. Mitochondrial Ca^2+^ levels were determined by flow cytometry (**d**), and MFI was shown (**e**). **f**, **g** HS-induced hyperpolarization of the mitochondrial membrane potential was determined by flow cytometry (**f**), and the ratio of JC-1 polymer/monomer was shown (**g**)**. h**, **i** CsA reversed HS-induced mPTP opening. The mPTP level was determined by flow cytometry (**h**), and MFI was shown (**i**). **j** Cytosolic mtDNA was decreased after treatment with CsA (*n* = 6). **k**–**q** HS-induced activation of the cGAS-STING pathway was attenuated by CsA. CsA inhibited HS-induced activation of STING and p-TBK1 and p-IRF3 (*n* = 3) (**k**–**n**, **p**). CsA inhibited HS-induced upregulation of CXCL10 (**o**) and IFN-β (**q**) (*n* = 6). **r** siRNA-mediated BAX knockdown did not affect the cGAS-STING pathway. The data were shown as the mean ± SD. *P < 0.05, **P < 0.01, ***P < 0.001

The release of mtDNA into the cytosol was identified in HS-HCE (Fig. 4a, b), but how HS induced mtDNA release into the cytosol is still unknown. mtDNA is released into the cytoplasm as a result of increased mitochondrial permeability, which may be mediated by mPTP^43^ or BAX.^33^ We speculated that mitochondrial permeability could increase under HS conditions, as mitochondrial changes (altered mitochondrial calcium levels and decreased mitochondrial membrane potential) have been observed in HS-HCE.^44,45^ We showed that HS induced morphological changes in mitochondria (loss of mitochondrial cristae and swelling in the mitochondrial matrix) in HCE (Fig. 5a). In addition, mPTP opening (Fig. 5b, c), an increase in mitochondrial Ca^2+^ levels (shown as Rhod-2 AM) (Fig. 5d, e) and hyperpolarization of the mitochondrial membrane potential (Δψ) (shown as JC-1) (Fig. 5f, g) were observed in HS-HCE.

To explore the role of mPTP in the activation of the cGAS-STING pathway, cyclosporin A (CsA), a mPTP inhibitor, was used to block mPTP opening.^46,47^ HS-induced mPTP opening was reversed (Fig. 5h, i), and mtDNA leakage was inhibited (Fig. 5j) in HCE cultured with CsA (5 µg/ml). Furthermore, treatment with CsA led to the downregulation of STING, p-TBK1, p-IRF3, CXCL10 and IFN-β (Fig. 5k–q).

It has been reported that mtDNA is released from mitochondria through BAX and that BAX is involved in dry eye.^48^ Hence, we further explored the association of BAX with the cGAS-STING pathway using siRNA. However, the HS-mediated upregulation of STING and CXCL10 could not be attenuated by BAX knockdown (Fig. 5r), suggesting that mtDNA release into the cytosol and activation of the cGAS-STING pathway were mediated through mPTP opening rather than the formation of BAX macropores. Taken together, our results demonstrated that cGAS-STING pathway activation in HS-HCE was determined by mPTP opening-mediated mtDNA leakage.

## Discussion

Innate immune responses hold a crucial function in the pathophysiology of ocular surface diseases and can be activated by multiple stresses.^16,49–51^ Mouse ocular surfaces treated with BAC and HCE exposed to hyperosmolarity are commonly used models to simulate ocular surface stresses in vivo and in vitro.^52,53^ Here, we demonstrated that environmental stresses induced mitochondrial dysfunction and mtDNA translocated into the cytosol, which initiated innate immune-inflammatory responses through activation of the ocular surface cGAS-STING pathway.

Innate immune response mediated by the cGAS-STING pathway is involved in a variety of inflammation-related diseases, such as AMD,^27^ Sjogren’s syndrome,^54^ systemic lupus erythematosus (SLE),^55,56^ Parkinson’s disease^28^ and AKI.^33^ By sensing cytoplasmic dsDNA, the cGAS-STING pathway is activated and initiates downstream inflammatory cytokines (CXCL10, IL-6, IL-8 and type I interferon).^33,56^ Innate immune responses play crucial roles in ocular surface diseases,^57–61^ but the role of the cGAS-STING pathway in ocular surface diseases exposed to various environmental stresses remains largely unexplored. In our study, we found that the cGAS-STING pathway was elevated in in vivo mouse model (Fig. 1e and Supplementary Fig. 1d), dry eye patients (Supplementary Fig. 2b), and an in vitro cell culture model (Fig. 3a, b). Activation of the cGAS-STING pathway induced the expression of CXCL10, IL-6 and IFN-β (Fig. 1g, 3d), which were reported to be increased in dry eye patients and dry eye mice,^62–67^ and CXCL10 could potentially serve as a biomarker of dry eye.^68^

Inhibition of the cGAS-STING pathway reduces the release of inflammatory molecules and the development of inflammation-related diseases.^28,30,31,33,43^ Hiroshi Maekawa et al. found that the cGAS-STING pathway triggered inflammatory responses and AKI progression, and genetic knockout of STING ameliorated inflammatory responses and AKI progression.^33^ Danielle A Sliter et al. showed that inflammation mediated by STING is triggered by mutations in parkin and PINK1. Consequently, this leads to the degeneration of dopaminergic neurons in the substantia nigra pars compacta and the development of motor impairments, ultimately causing the early-onset of Parkinson’s disease. However, it has been found that the adverse effects mentioned above can be mitigated through the inhibition of STING.^28^ Our results demonstrated that ocular surface damage and associated inflammation could be prevented by either knockdown or pharmacological suppression of STING (Figs. 1h–m, 2a–h), indicating the potential of the cGAS-STING pathway as a therapeutic target for ocular surface diseases.

Activation of the cGAS-STING pathway depends on the recognition of intracellular nucleic acids,^23^ including cytoplasmic mtDNA or oxidized mtDNA.^42,69^ A previous study reported that HS mediated mtDNA oxidation in an in vitro dry eye model.^40^ However, oxidized mtDNA was not the main agent responsible for cGAS-STING pathway activation in dry eye in this study, since inhibiting mtDNA oxidation by exogenous 8-OHDG did not downregulate the cGAS-STING pathway (Fig. 4i, j). In contrast, HS promoted mtDNA translocated into the cytosol and caused cGAS-STING pathway activation. Exogenous introduction of mtDNA into the cytoplasm promoted cGAS-STING pathway activation, while inhibition of mtDNA translocated into the cytosol alleviated it (Fig. 4c–h). These results suggested that mtDNA translocated into the cytosol, rather than mtDNA oxidation, caused the activation of the cGAS-STING pathway to further mediate inflammation.

Mitochondrial homeostasis can maintain corneal epithelial function, resist oxidative damage and inflammation, and promote corneal epithelial migration and repair.^70^ However, corneal epithelial cells are vulnerable to mitochondrial dysfunction due to their high exposure to multiple environmental stresses.^71^ HS can change the levels of mitochondrial calcium and depolarize the mitochondrial membrane potential,^44^ which causes ROS accumulation^72^ and BAX expression.^48^ Consistent with previous studies, we found that HS induced mitochondrial dysfunction (Fig. 5a–g). In addition, HS can induce mPTP opening, which increases mitochondrial permeability.^73^ Increased BAX expression also leads to an increase in mitochondrial membrane permeability.^74^ Therefore, we further explored the contribution of the mPTP and BAX to the promotion of mtDNA release and activation of the cGAS-STING pathway.^33,43^ Knockdown of BAX did not inhibit STING expression in our study (Fig. 5r). In contrast, we observed mPTP opening under HS exposure (Fig. 5b, c), and inhibition of this mPTP opening reduced cytoplasmic DNA and downregulated the cGAS-STING pathway (Fig. 5j–q). These results indicated that HS induced mtDNA leakage through mPTP.

In summary, in this study, we demonstrated that HS induced mitochondrial dysfunction and mPTP opening, which led to mtDNA leakage. Cytoplasmic mtDNA further activated the cGAS-STING pathway to mediate ocular surface inflammation. Hence, we identified a new mechanism of inflammation activation in ocular surface exposure to environmental stresses. Therapeutic strategies that target this pathway have the potential to prevent or treat ocular surface diseases, such as dry eye.

## Materials and methods

### Participant recruitment and clinical evaluation

A total of 26 dry eye patients and 22 normal subjects were recruited from Xiamen University affiliated Xiamen Eye Center. Dry eye patients were selected based on the following inclusion criteria: tear breakup time (TBUT) ≤ 5 s, or CFS score ≥ 1. The normal subjects consisted of individuals without any ocular disorders. The demographic information and ocular sign scores for all participants can be found in Table S1. Using the Schirmer’s I test, the tear samples were collected. The Schirmer strips were placed in 0.6 mL centrifuge tubes and maintained at a temperature of −80 °C until further analysis. The entire research procedure adhered to the principles outlined in the Declaration of Helsinki and was approved by the Medical Ethics Committee of Xiamen University (XDYX202209K23). Written informed consent was obtained from all participants.

### Reagent and antibodies

C-176, ethidium bromide (EtBr) and cyclosporin A (CsA) were obtained from Selleck (Houston, TX, USA). 8-OHDG was obtained from MCE (China). Benzalkonium chloride (BAC) was obtained from Sigma‒Aldrich (Louis, MO, USA). Primary antibodies against dsDNA, cGAS, STING, TBK1, p-TBK1, IRF3 and p-IRF3 were obtained from Proteintech (Chicago, IL, USA), Abcam (Cambridge, UK) or CST (Danvers, MA, USA). The HRP-conjugated secondary antibody was purchased from Invitrogen (Carlsbad, CA, USA) (Table S3).

### Extraction of tear proteins from Schirmer strips

Tear proteins were extracted from Schirmer strips following the previously described method.^75^ To initiate the extraction, RIPA buffer was added to a 0.6 mL centrifuge tube and incubated for 30 min. Subsequently, the tube was punctured in the center of the bottom, and the buffer was afterwards transferred to a 1.5 mL centrifuge tube, and centrifuged for 10 min at 12,000 rpm (Fig. 1a). The protein concentration in the collected fluid was quantified using a BCA protein assay kit (Catalog No. 23225; Thermo Fisher Scientific, MA, USA).

### Animal model

STING^−/−^ mice (C57BL/6J background) were purchased from the Model Animal Research Center of Nanjing University and Gempharmatech Company (gene ID, T012747). Through PCR analysis of tail DNA (data not shown), the successful deletion of the targeted genes was confirmed. Female (8–10 weeks old) wild-type (WT) or STING^-/-^ mice were used for the experiments. Following the previously described method,^52^ ocular damage induced by BAC was performed. Briefly, a solution containing 0.075% BAC (5 μL) was applied to both eyes twice daily for 7 consecutive days, while control mice received 5 µL of PBS. Different concentrations of C-176 (ranging from 1 to 100 µM) were administered four times daily to both eyes, starting 10 min after BAC treatment. The dry eye model induced by extraorbital lachrymal gland excision (ELGE) was conducted as previously described.^36^ All mice were housed in a controlled environment with a constant temperature (22–26 °C), relative humidity of 60% ± 10%, and a 12-h cycle alternating between light and darkness (8:00 a.m. to 8:00 p.m.). All procedures conducted in the experiment followed the guidelines outlined by the Association for Research in Vision and Ophthalmology (ARVO) Statement for the Utilization of Animals in Ophthalmic and Vision Research and obtained approval from the Animal Ethics Committee of Xiamen University (Approval ID: XMULAC20180053).

### Measurement of ocular surface damage

CFS and PAS staining were employed to quantify the extent of ocular surface damage. CFS was evaluated following the previously described protocol.^76^ Goblet cells in paraffin sections were stained using the Periodic Acid-Schiff (PAS) reagent (Catalog No. 395B-1KT; Sigma-Aldrich, Louis, USA). The superior and inferior conjunctival images were captured and the density of goblet cells was quantified using NIS Elements software.

### Cell culture

Immortalized HCE were cultured following the previously reported method.^77^ Cells at passages 3–5 were cultivated in a hyperosmotic medium containing 500 mOsM by supplementing with 96 mM NaCl. To deplete mitochondrial DNA (mtDNA), HCE were treated with 0.2 μg/mL EtBr for 48 h. Subsequently, HCE were incubated with 8-OHDG (5 µM) or CsA (5 µg/mL) for 24 h.

### RNA extraction and quantitative real-time PCR

Total RNA from HCE or mouse cornea was isolated using the PicoPure RNA isolation kit (Arcturus, Mountain View, CA, USA). Quantitative real-time PCR was conducted following the previously reported protocol.^52^ All primer sequences are listed in Table S2.

### Western blotting

Lysates were prepared from cellular samples and mouse corneal tissues by employing RIPA buffer containing Halt protease and phosphatase inhibitor cocktails. The concentration of total protein in the lysates was determined using a BCA Protein Assay Kit (Thermo Fisher Scientific, Waltham, MA, USA). The proteins were separated on an SDS polyacrylamide gel and subsequently transferred to PVDF membranes. Subsequently, 5% bovine serum albumin (BSA) in Tris-buffered saline with 0.2% Tween-20 (TBST) was utilized to block the membranes for 1 h at room temperature. Following this, primary antibodies against cGAS, STING, TBK1, p-TBK1, IRF3, and p-IRF3 were incubated with the membranes overnight at 4 °C. After three washes with TBST, the membranes were incubated with an HRP-conjugated IgG antibody, diluted to 1:10,000, for 1 h at room temperature. Finally, the antigen was detected using a commercial imaging system (Molecular Imager ChemiDoc XRS; Bio-Rad Laboratories, CA, USA).

### RNA interference

In this study, siRNAs specifically targeting cGAS, STING, and BAX were obtained from Hanbio (Shanghai, China). The transfection of these siRNAs into HCE cells was performed using LipoFiter™ Liposomal Transfection Reagent (Hanbio, Shanghai, China), following the instructions provided by the manufacturer. As a negative control, a nonspecific siRNA provided by the same supplier was used. After transfection, the cells were stimulated according to the experimental requirements. The specific siRNA sequences used in this study are listed in Table S2.

### DNA isolation and mtDNA copy number analysis

To extract cytosolic DNA, the following procedure was carried out, as previously described.^33^ HCE exposed to hyperosmotic medium for 24 h were divided into two equal aliquots. Resuspend the first aliquot in 500 μL of DNA extraction buffer containing 5 mM EDTA (pH 8.0), 0.2% SDS, 100 mM Tris-HCl (pH 8.5), 200 mM NaCl, and 100 μg/mL proteinase K. This resulting extract was used as a normalized control for the total amount of mtDNA. Resuspend the second aliquot in 500 μL buffer composed of 25 mg/mL digitonin, 150 mM NaCl, and 50 mM HEPES (pH 7.4). Cell membranes were then permeabilized by incubation at room temperature for 10 min. Following this, the sample was centrifuged at 1000 × *g* for 10 min to pellet intact cells. The supernatant containing the cytosol was carefully transferred to a new tube and then subjected to further centrifugation at 17,000 × *g* for 10 min to remove any remaining cellular debris. The DNA present in the cytosolic fraction was isolated using a DNA isolation kit from Tiangen (Beijing, China). Real-time PCR was performed on DNA (10 ng) using the SYBR qPCR master mix on the LightCycle96 system (Bio-Rad). (Vazyme). MtDNA (ND1, ND2, ATP6, CO2, and DLOOP) and nuclear DNA (18S) contents were amplified for determination. The mtDNA/nDNA ratio was calculated to determine the mtDNA copy number. Refer to Table S2 for the specific primer sequences used in this study.

### mtDNA isolation and transfection

HCE specimens were utilized for the isolation of mtDNA employing a mitochondrial DNA isolation kit (BioVision) as per the prescribed protocol. The obtained mtDNA was reconstituted in TE buffer and maintained at a temperature of −20 °C for potential utilization. HCE cells were then seeded in six-well plates and subjected to transfection with mtDNA (1 μg/well) via the application of Lipofectamine 3000 (Thermo Fisher Scientific) following the instructions provided by the manufacturer.

### Mitochondrial membrane potential assay

HCE cells exposed to hyperosmotic medium for 24 h were collected, and a mitochondrial membrane potential assay kit with JC-1 (Beyotime, Shanghai, China) was employed to examine the potential of the mitochondrial membrane. Following staining, the stained cells were subjected to analysis using a Beckman Cytoflex S flow cytometer (Beckman, USA).

### Mitochondrial permeability transition pore assay

The levels of mPTP were assessed following a 24-h culture in hyperosmotic medium. To perform this assessment, a mitochondrial permeability transition pore assay kit from Yeasen (Shanghai, China) was utilized, following the provided instructions by the manufacturer. Stained cells were analyzed using a Beckman Cytoflex S flow cytometer.

### Detection of mitochondrial Ca^2+^ levels

HCE cells were carefully washed three times with Hank’s balanced salt solution (HBSS) and subsequently incubated with Rhod-2 AM (4 µM, Yeasen, Shanghai, China) for 60 min at 37 °C. Following the incubation, the cells underwent another set of three washes with HBSS and were further incubated for an additional 30 min at 37 °C. The intensities of Ca^2+^ were detected by flow cytometry (Beckman Cytoflex S, USA). FCM data were plotted and quantified with Flowjo software (Treestar).

### Transmission electron microscopy

HCE cells were meticulously immobilized for a duration of one night at a temperature of 4 °C in a solution incorporating 2.5% glutaraldehyde and 150 mM sodium cacodylate (pH 7.4). Following the initial fixation, the cells underwent a sequential process involving further fixation using 1% OsO_4_, staining with uranyl acetate, dehydration utilizing ethanol, and ultimately being encased in epoxy resin. Imprints of exceptionally thin sections were then treated with uranyl acetate and lead citrate after being situated on formvar-coated grids. Ultimately, a Hitachi HT-7800 transmission electron microscope was employed to capture the visual representations.

### Immunofluorescence analysis

HCE cells were subjected to hyperosmotic medium treatment for a duration of 24 h. Subsequently, the cells were incubated with 100 nM MitoTracker Red CMXRos for 30 min. Following the incubation, the cells were washed twice with PBS, and afterwards fixed and permeabilized by utilizing 0.1% Triton X-100 for 20 min. Following the application of 2% BSA to block non-specific binding, the cells were incubated with dsDNA (diluted 1:200 in 1% BSA) overnight at 4 °C. For signal detection, a secondary antibody conjugated with Alexa Fluor 488 was utilized. Finally, a confocal microscope (Zeiss LSM 880 Airyscan, Germany) was employed to captured the images.

### Measurement of cGAMP

Following the manufacturers’ instructions of cGAMP ELISA kit (Cayman Chemical), the cGAMP levels in the cell lysate was determined. The result was normalized to the total protein concentration.

### Statistical analysis

Data were analyzed using the Mann‒Whitney *t* test or one-way analysis of variance. The correlation of the two numerical variables was tested by Spearman analysis. A value of *P* < 0.05 was considered statistically significant. Using SPSS 22.0 software and GraphPad Prism version 7 software (GraphPad Software, CA, USA), calculations were performed.

### Supplementary information

Supplementary Figures and Tables

## Data Availability

The corresponding authors made the data utilized in the present investigation accessible to interested individuals upon a reasonable request.
