# Supplementary material for: The cGAS-STING pathway-dependent sensing of mitochondrial DNA mediates ocular surface inflammation
Source: Signal Transduct Target Ther. 2023 Sep 21;8:371. doi: 10.1038/s41392-023-01624-z (PMC10514335; doi:10.1038/s41392-023-01624-z)
Supplement: Supplementary file 1 — Supplementary Figures and Tables [file 41392_2023_1624_MOESM1_ESM.docx]

Supplementary Materials for

The cGAS-STING Pathway-Dependent Sensing of Mitochondrial DNA Mediates Ocular Surface Inflammation

Weijie Ouyang^1,2,#^, Shoubi Wang^1,3,#^, Dan Yan^1,2^, Jieli Wu^4^, Yunuo Zhang^1,2^, Wei Li^1,2^ Jiaoyue Hu^1,2,*^, and Zuguo Liu^1,2,5,*^

**Corresponding author:**

Zuguo Liu, Eye Institute of Xiamen University, 168 Daxue Road, Xiamen, Fujian 361005, P.R. China. E-mail: zuguoliu@xmu.edu.cn.Phone: 86-592-218-3761

Jiaoyue Hu, Eye Institute of Xiamen University, 168 Daxue Road, Xiamen, Fujian 361005, P.R. China. E-mail: mydear_22000@163.com. Phone: 86-592-218-3761

^#^ These authors contributed equally to this work.

**This PDF file includes:**

Supplementary figures. S1 to S2

Tables S1 to S3


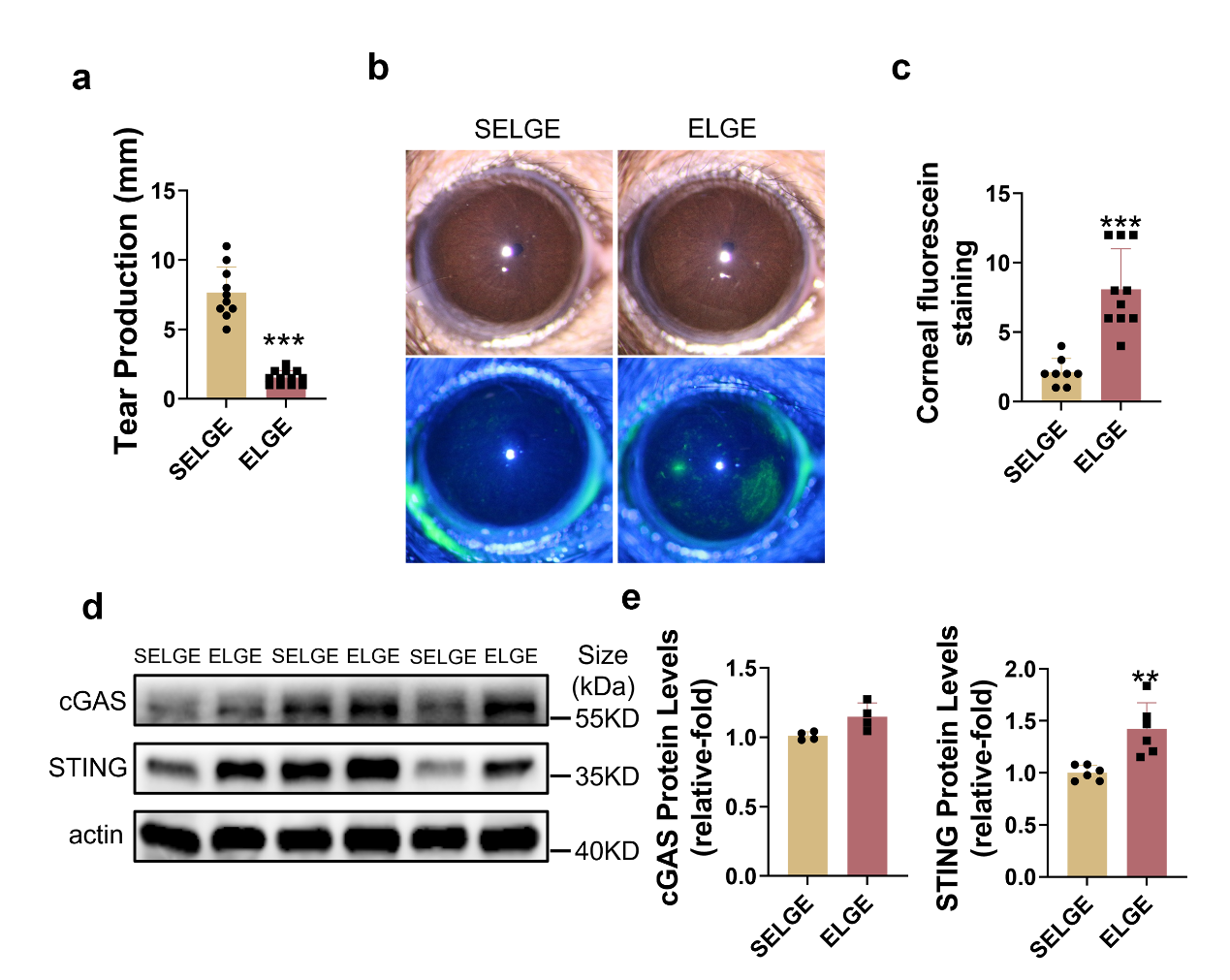


Supplementary Figure. 1.

**The cGAS-STING pathway was activated in ELGE mice. a.** The volume of tear production in sham extraorbital lachrymal gland excision (SLEGE) and extraorbital lachrymal gland excision (ELGE) mice. **b, c.** Increased CFS scores in ELGE mice. Representative images of CFS **(b)** and mean scores **(c)** in SLEGE and ELGE mice are shown. **d, e.** cGAS and STING protein levels were increased in ELGE mice (n=4 or 6). The data were shown as the mean ± SD. **P < 0.01.


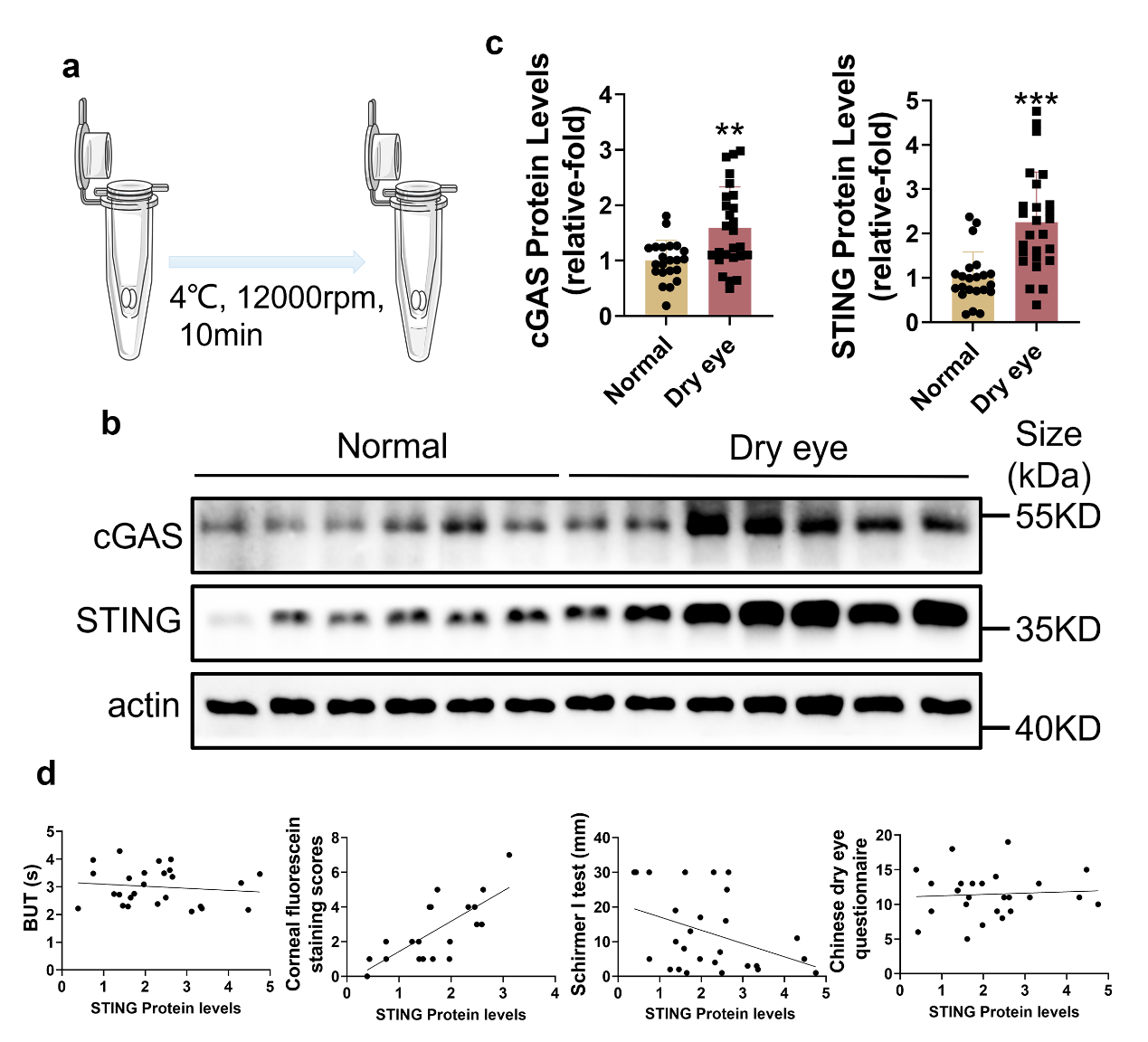


Supplementary Figure. S2.

**Activation of the cGAS-STING pathway was observed in tears of dry eye patients.** **a.** Schematic illustration of tear protein extraction. **b.** The expression of cGAS and STING in normal subjects and dry eye patients. **c.** Statistical analysis of cGAS and STING protein levels in normal subjects and dry eye patients. The data were shown as the mean ± SD. **P < 0.01, ***P <0.001. n=22 or 26. **d.** The correlation between STING protein levels and TBUT, CFS scores, Schirmer I test and Chinese dry eye questionnaire.

Table S1. Characteristics of the normal and dry eye subtypes

|  | | | | | | |
| --- | --- | --- | --- | --- | --- | --- |
| Group | Gender (M/F) | Age | TBUT (s) | CFS scores | Schirmer’s I Test (mm) | Chinses dry eye questionnaire |
| Normal | 10/12 | 22.77±2.84 | 8.45±3.05 | 0.34±0.91 | 26.38±6.49 | 4.46±2.67 |
| Dry eye | 12/14 | 25.27±2.34 | 4.52±2.30 | 2.10±2.22 | 11.23±10.64 | 11.04±3.69 |

Table S2. Oligonucleotides

|  | | |
| --- | --- | --- |
| human-STING-F | AGCATTACAACAACCTGCTACG | This paper |
| human-STING-R | GTTGGGGTCAGCCATACTCAG | This paper |
| human-cGAS-F | ACATGGCGGCTATCCTTCTCT | This paper |
| human-cGAS-R | GGGTTCTGGGTACATACGTGAAA | This paper |
| human-IFN-β-F | GACCAACAAGTGTCTCCTCCAAA | This paper |
| human-IFN-β-R | AGCAAGTTGTAGCTCATGGAAAGAG | This paper |
| human-BAX-F | TGGAGCTGCAGAGGATGATTG | This paper |
| human-BAX-R | CCCAGTTGAAGTTGCCGTCAG | This paper |
| human-CXCL10-F | AGTGGCATTCAAGGAGTACC | This paper |
| human-CXCL10-R | TGATGGCCTTCGATTCTGGA | This paper |
| human-actin-F | CATGTACGTTGCTATCCAGGC | This paper |
| human-actin-R | CTCCTTAATGTCACGCACGAT | This paper |
| mouse-STING-F | CTACATTGGGTACTTGCGGTT | This paper |
| mouse-STING-R | GCACCACTGAGCATGTTGTTATG | This paper |
| mouse-cGAS-F | GTCGGAGTTCAAAGGTGTGGA | This paper |
| mouse-cGAS-R | GACTCAGCGGATTTCCTCGTG | This paper |
| mouse-CXCL10-F | AAGTGCTGCCGTCATTTTCT | This paper |
| mouse-CXCL10-R | GTGGCAATGATCTCAACACG | This paper |
| mouse-IL-6-F | CCACTTCACAAGTCGGAGGC | This paper |
| mouse-IL-6-R | GCCATTGCACAACTCTTTTCTCA | This paper |
| mouse-actin-F | GTGACGTTGACATCCGTAAAGA | This paper |
| mouse-actin-R | GCCGGACTCATCGTACTCC | This paper |
| Mouse STING KO-F | ACCTGATGGGAGGTATCTACCGG |  |
| Mouse STING KO-R | CCAGCAACTAGCATCAGAACCTCC |  |
| Mouse STING WT-F | GGTGCCTGACAACCTGAGTGTAG |  |
| Mouse STING WT-R | CCTCAATGCTCTCATAGCCTTCAC |  |
| human-mt-ND1-F | CTCTTCGTCTGATCCGTCCT | ^1^ |
| human-mt-ND1-R | TGAGGTTGCGGTCTGTTAGT | ^1^ |
| human-mt-ND2-F | GTAGACAGTCCCACCCTCAC | ^1^ |
| human-mt-ND2-R | TTGATCCCGTTTCGTGCAAG | ^1^ |
| human-mt-ATP6-F | AATCCAAGCCTACGTTTTCACA | ^2^ |
| human-mt-ATP6-R | AGTATGAGGAGCGTTATGGAGT | ^2^ |
| human-mt-CO2-F | AATCGAGTAGTACTCCCGATTG | ^2^ |
| human-mt-CO2-R | TTCTAGGACGATGGGCATGAAA | ^2^ |
| human-mt-DLOOP-F | CTATCACCCTATTAACCACTCA | ^2^ |
| human-mt-DLOOP-R | TTCGCCTGTAATATTGAACGTA | ^2^ |
| human-mt-18S-F | TAGA GGGACAAGTGGCGTTC | ^3^ |
| human-mt-18S-R | CGCTGAGCCAGTCAGTGT | ^3^ |
| human-siRNA-cGAS-F | AUCUAUUCUCUAGCAACUUAATT | This paper |
| human-siRNA-cGAS-R | UUAAGUUGCUAGAGAAUAGAUTT | This paper |
| human-siRNA-STING-F | GCCCGGAUUCGAACUUACAAUTT | This paper |
| human-siRNA-STING-R | AUUGUAAGUUCGAAUCCGGGCTT | This paper |
| human-siRNA-BAX-F | ACAUGUUUUCUGACGGCAA | This paper |
| human-siRNA-BAX-R | UUGCCGUCAGAAAACAUGU | This paper |
| human-siRNA-NC-F | UUCUCCGAACGUGUCACGUTT | This paper |
| human-siRNA-NC-R | ACGUGACACGUUCGGAGAATT | This paper |

Table S3. Antibodies and Reagents

|  | | |
| --- | --- | --- |
| Antibodies |  |  |
| cGAS | Abcam | ab252416 |
| cGAS | cell signaling technology | 15102 |
| STING | Proteintech | 19851-1-AP |
| TBK1 | cell signaling technology | 3504 |
| p-TBK1 | cell signaling technology | 5483T |
| IRF3 | Abcam | ab68481 |
| p-IRF3 | Abcam | ab76493 |
| dsDNA | Abcam | ab27156 |
| Reagents |  |  |
| EtBr | Selleck | S3689 |
| C-176 | Selleck | S6575 |
| cyclosprin A | Selleck | S2286 |
| 8-OHDG | MCE | HY-W011540 |
| JC-1 | BEYOTIME | C2006 |
| Rhod-2, AM, | Yeasen | 40776ES50 |
| mPTP kit | Yeasen | 40756ES60 |
| Mito Tracker | Solarbio | M9940 |
| Mito DNA Isolation Kit | Biovision | K280-50 |

Reference

1 Yu, C. H. *et al.* TDP-43 Triggers Mitochondrial DNA Release via mPTP to Activate cGAS/STING in ALS. *Cell* **183**, 636-649 e618 (2020).

2 Aarreberg, L. D. *et al.* Interleukin-1beta Induces mtDNA Release to Activate Innate Immune Signaling via cGAS-STING. *Mol Cell* **74**, 801-815 e806 (2019).

3 Aguirre, S. *et al.* Dengue virus NS2B protein targets cGAS for degradation and prevents mitochondrial DNA sensing during infection. *Nat Microbiol* **2**, 17037 (2017).
